# Evaluation of the effect of carrier material on modification of release characteristics of poor water soluble drug from liquisolid compacts

**DOI:** 10.1371/journal.pone.0249075

**Published:** 2021-08-02

**Authors:** Beenish Ali, Amjad Khan, Hamad S. Alyami, Majeed Ullah, Abdul Wahab, Munair Badshah, Attiqa Naz

**Affiliations:** 1 Department of Pharmacy, Abasyn University, Peshawar, Pakistan; 2 Department of Pharmacy, Kohat University of Science and Technology (KUST), Kohat, Pakistan; 3 Department of Pharmaceutics, Najran University, Najran, Saudi Arabia; 4 Islam College of Pharmacy, Sialkot, Pakistan; ISF College of Pharmacy, Moga, Punjab, India, INDIA

## Abstract

Liquisolid compact is a novel dosage form in which a liquid medication (liquid drug, drug solution/dispersion in non-volatile solvent/solvent system) is converted to a dry, free flowing powder and compressed. Objective of the study was to elucidate the effect of carrier material on release characteristics of clopidogrel from liquisolid compacts. Different formulations of liquisolid compacts were developed using microcrystalline cellulose, starch maize, polyvinyl pyrollidone and hydroxypropyl methylcellulose as carrier material in three concentrations (40, 30 and 20%, w/w). Liquid vehicle was selected on the basis of solubility of clopidogrel. Colloidal silicondioxide was used as coating material and ratio of carrier to coating material was kept 10. A control formulation comprised of microcrystalline cellulose (diluents), tabletose-80 (diluents), primojel (disintegrant) and magnesium stearate (lubricant) was prepared by direct compression technique and was used for comparison. All the formulations were evaluated at pre and post compression level. Acid solubility profile showed higher solubility in HCl buffer pH2 (296.89±3.49 μg/mL). Mixture of propylene glycol and water (2:1, v/v) was selected as liquid vehicle. Drug content was in the range of 99–101% of the claimed quantity. All the formulations showed better mechanical strength and their friability was within the official limits (<1%). Microcrystalline cellulose and starch maize resulted in faster drug release while polyvinyl pyrollidone and HPMC resulted in sustaining drug release by gel formation. It is concluded from results that both fast release and sustained release of clopidogrel can be achieved by proper selection of carrier material.

## Introduction

**Fig 1:**
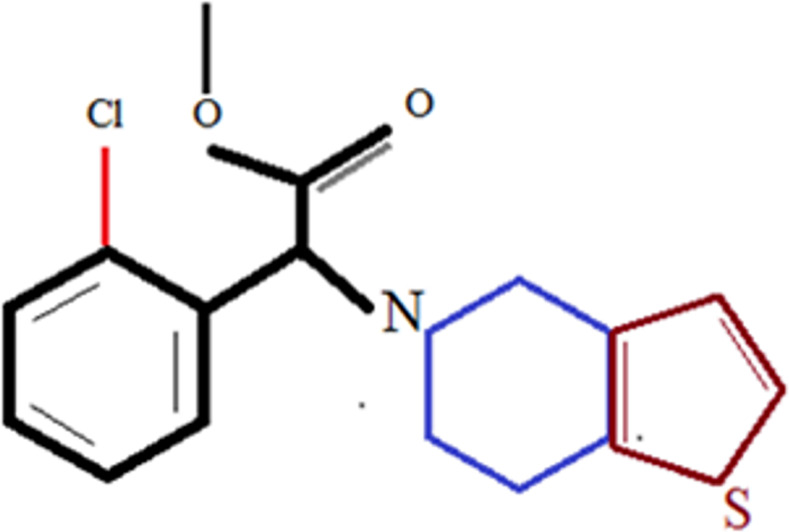
Structural formula of clopidogrel.

The rate and extent of drug absorption from the gastrointestinal (GI) tract is very intricate and affected by many factors, including physicochemical factors, physiological factors, and factors related to the dosage form [1]. Despite this complexity, the Biopharmaceutics Classification System (BCS) [2] revealed that the essential key parameters controlling oral drug absorption are the solubility/dissolution of the drug dose in the GI milieu and the permeability of the drug through the GI membrane. Any modification in release characteristics of the drug will change pharmacokinetics and subsequent therapeutic effect of the drug. A number of techniques like, solid dispersion [3], liquisolid compact [4], complexation [5], micronization [6] and salt formation [7], have been applied to improve the dissolution rate of poorly soluble drugs. In comparison to other dosage forms that influence bioavailability, liquisolid compacts provide many additional advantages, for example, lower production costs, final processing similar to tablets or hard capsules, minimized pH influence on dissolution rate, and the possibility to prepare dosage forms with controlled drug delivery [4]. In liquisolid compact, liquid medication (liquid drug or drug solution in nonvolatile solvent) is converted to a free flowing, dry and compressible powder. Liquisolid compacts are prepared by absorbing liquid medication on a carrier material and it’s blending with other ingredients to prepare compressed tablet [8]. In the liquisolid systems, even though the drug is presented as solid dosage form, it is held within the powder substrate in solution or in almost molecularly dispersed state. Therefore, due to their significantly increased wetting properties and surface area of drug available for dissolution, liquisolid compacts of water insoluble substances are expected to display enhanced drug release characteristics and consequently improved oral bioavailability [9]. Liquisolid compacts were pioneered by Spireas and Sadu [10] and Spireas et al. [11], where the dissolutions of pridinsolone and hydrocortisone were improved by using the liquisolid technique. Since then, extensive research has been carried out to improve dissolution rate of many BCS class-II and class-IV drugs [9, 12–17]. Liquisolid systems were initially designed to improve drug dissolution rate and was mainly applied for low dose drugs like meloxicam, olmesartan and telmisartan. Recently, it was investigated as a possible means to sustain the drug release by proper selection of carrier excepients. Sustained release dosage forms are designed to release the drug at a predetermined rate by maintaining a constant drug release for specific period of time with minimum side effects in terms of efficacy, safety and patient compliance. So far, only few drugs, to the best of our knowledge, have been formulated as liquisolid systems with prolonged drug release [18–20]. Most of the reported sustained release liquisolid formulations are based on hydrophobic carriers such as Eudragit® RL or RS instead of hydrophilic carriers, or hydrophilic carrier with the incorporation of retarding agents. Hydrophobic carriers may lead to poor wetting properties of the compacts resulting in slow disintegration and thus, prolonged drug release. In the presented study, it is hypothesized that along with enhancing dissolution rate, liquisolid compacts can also sustain drug release by proper selection of hydrophilic carrier material. In this study clopidogrel (CLP) was selected as model drug for formulation development of liquisolid compacts. CLP is methyl (S)- α-(2-chlorophenyl)-6,7-dihydrothieno[3,2-c]pyridine-5(4H)-acetate, having a molecular weight 321.82 g/mol [21–23]. Partition co-efficient of CLP is 3.9 at pH 7.4 in a water/octanol medium and has a pKa value of 4.55 [21]. It has pH dependent solubility and decrease in pH, increases its solubility [23]. Structural formula of clopidogrel is given in Fig 1. Clopidogrel is an antiplatelet drug available as tablet dosage form (75 mg) alone and in combination with other drugs like aspirin [24]. It was discovered in 1986 and approved in occidental countries in 1997 [25]. This compound has been demonstrated to prevent cardiovascular death in atherosclerotic patients in CAPRIE, a randomized phase III, triple-blinded clinical trial enrolling more than 19,000 patients with atherosclerotic disease [26, 27].

It was objective of the study to modify (enhance and sustain) the *in vitro* release of clopidogrel by liquisolid compacts technique using different carrier material and elucidate the effect of carrier material on dissolution rate. In the present study, various formulations of liquisolid compacts were developed, using different carrier material and their effect on release rate of clopidogrel was evaluated.

## Material and methods

### Material and instrumentation

The model drug clopidogrel (Mithri Laboratories Ltd. India; purity 99.81% with reference to USP standard) was obtained from Ferozsons Laboratories Ltd. Nowshera, Pakistan. Excipients used in the study, included tablettose-80 (Molkerei Meggle, Germany), microcrystalline cellulose (F.M.C International, Ireland), primojel {sodium starch glycolate} (F.M.C International, Ire Land), Colloidal silicon dioxide {Aerosil-200} (F.M.C International, Ireland), hydroxylpropyl methylcellulose (Merck KGA, Germany), polyvinyl pyrollidone (I.S.P. Technology, Texas), starch maize (I.C.I, Pakistan) and magnesium stearate (Coin Powder International Company Ltd, Taiwan), were purchased from local market of Peshawar, Pakistan. All the excipients were of pharmaceutical grade and were used as received.

Equipments used for preparation of liquisolid compact, included digital balance (Libror AEG-120, Shimadzu, Japan), laboratory scale double cone mixer (Morgan Machinery Ltd, Pakistan) and commercial scale rotary tablet compression machine ZP-19 (S.T.C. China). Tablet hardness and thickness tester, (Pharma Test, Germany), tablet disintegration tester (Pharma Test, Germany) and dissolution testing apparatus (Pharma Test, Germany) were used for evaluation of liquisolid compacts. Purified water was prepared by Milli-Q® system (Millipore, Milford, MA, USA).

### Determination of solubility of clopidogrel in different non-volatile solvent systems

Flask shaking method was applied for determination of solubility of clopidogrel [28]. Solubility of the clopidogrel was determined in following non-volatile solvents and solvent systems;

- Water
- Glycerine
- Propylene glycol (PG),
- PG + water (1:1)
- PG + water (1:2)
- PG + water (2:1)
- PG + water +Tween-60 (0.5%)
- Glycerin + Water (1:1)
- Glycerin + Water (1:2)
- Glycerin + Water (2:1)
- Glycerin + Water + Tween-60 (0.5%)

Solubility was calculated using Eq 1;

$$Solubility\;of\;CLP=\frac{Quantity\;of\;CLP(mg)}{Volume\;of\;solution(mL)}$$ (1)

### Stability of clopidogrel solution prepared in non volatile solvent system

For determination of stability of CLP in selected non-volatile solvent system, drug solution kept in a screw capped glass bottle at elevated conditions of temperature (45 ± 5°C) and humidity (75 ± 5% RH) for 10 days. Drug content of the solution was determined on daily basis and compared for variability. At each sampling point drug content was determined in triplicate and results were presented as mean ± standard deviation (n = 3). At each sampling point, samples were also evaluated for color and odor.

### Design of liquisolid compact

#### Optimization of quantity of liquid vehicle

On the basis of better solubility, mixture of propylene glycol and water (2:1 w/w) was selected as solvent system for formulation development of liquisolid compacts of CLP. Objective of the study was to evaluate the effect of carrier material on release of CLP from liquisolid compacts, so fixed quantity of solvent system was used throughout the study.

#### Mathematical modeling of liquisolid compacts

Several liquisolid formulations were prepared, having constant quantity of solvent system and different concentrations (40, 30 and 20% w/w) of the carrier material (microcrystalline cellulose, starch, polyvinyl pyrollidone (PVP k-120) and hydroxypropyl methylcellulose (HPMC)). Colloidal silicondioxide (aerocil) was used as coating material and ratio of carrier to coating material was kept constant at 10. Formulation parameters for liquisolid compacts were calculated on the basis of mathematical models proposed by Khan et al [29], as follows;

*Liquid load factor*. Liquid load factor is the ratio of weight of liquid medication (w) to the weight of carrier material (Q) [29]. Drug was dissolved in selected nonvolatile solvent by gentle heating on water bath (50 ± 2°C) to form a clear solution. Drug solution was added to the carrier material, in portions, by proper blending in a laboratory scale double cone mixer and kept un-disturbed for 15 min. Liquid load factor was calculated using following equation;

$$\text{Lf}=\frac{\text{W}}{\text{Q}}$$ (2)

Where,

W = weight of liquid medication
Q = weight of carrier material

Liquid load factor predicts the ability of the carrier material to withhold the liquid medication

*Excipients ratio*. Excipient ratio was calculated on the basis of weight of carrier and coating material [29], using following equation;

$$\text{R}=\frac{\text{Q}}{\text{q}}$$ (3)

Where,

Q = weight of carrier
q = coating material

Table 1 summarizes the design of liquisolid formulations, containing 75 mg of clopidogrel.

**Table 1:** Parameters for different formulations of liquisolid compacts of clopidogrel.

Formulation Code	Solvent System (g)	Drug Conc. In Solvent System (% w/w)	Carrier/ Coating (R)	Liquid Vehicle (g)	API (g)	Carrier (Q) (g)	Liquid Load Factor	Unit Dose (mg)
LCS-1	30	20	10	37.5	7.5	40	0.94	75
LCS-2	30	20	10	37.5	7.5	30	1.25	75
LCS-3	30	20	10	37.5	7.5	20	1.88	75
LCS-4	30	20	10	37.5	7.5	40	0.94	75
LCS-5	30	20	10	37.5	7.5	30	1.25	75
LCS-6	30	20	10	37.5	7.5	20	1.88	75
LCS-7	30	20	10	37.5	7.5	40	0.94	75
LCS-8	30	20	10	37.5	7.5	30	1.25	75
LCS-9	30	20	10	37.5	7.5	20	1.88	75
LCS-10	30	20	10	37.5	7.5	40	0.94	75
LCS-11	30	20	10	37.5	7.5	30	1.25	75
LCS-12	30	20	10	37.5	7.5	20	1.88	75

Data has been rounded off to two digits after decimal point.

### Preparation of liqui-solid compacts

Drug was dissolved in solvent system by gently heating at 50 ± 2°C with constant stirring until clear solution (liquid vehicle) was formed. Drug solution was slowly incorporated into carrier material with continuous blending. The mixture was kept un-disturbed for 15 min to completely absorb the liquid medication into core of the carrier material; coating material was added and blended for 5 min. Other excipients (disintegrant, lubricant and diluents) were added to the mixture, as per Table 2 and blended for further 5 min. All the formulations of liquisolid compacts were compressed using rotary compression machine (ZP-21, China) fitted with 12 mm round punches with beveled edges and bisection line. A control formulation was prepared by direct compression of the physical blend of drug and excipients used in preparation of the final liquisolid compacts. Control formulation comprised of microcrystalline cellulose (diluents), tabletose-80 (diluents), primojel (disintegrant) and magnesium stearate (lubricant).

**Table 2:** Composition of different formulations of liquisolid compacts containing clopidogrel.

Ingredients	LCS-01	LCS-02	LCS-03	LCS-04	LCS-05	LCS-06	LCS-07	LCS-08	LCS-09	LCS-10	LCS-11	LCS-12
Clopidogrel	9.04	9.04	9.04	9.04	9.04	9.04	9.04	9.04	9.04	9.04	9.04	9.04
Micro crystalline cellulose	48.19	36.144	24.096	–	–	–	–	–	–	–	–	–
Colloidal silicon dioxide	4.819	3.614	2.41	4.819	3.614	2.41	4.819	3.614	2.41	4.819	3.614	2.41
Starch	–	–	–	48.19	36.144	24.096	–	–	–	–	–	–
Poly vinyl pyrrolidine	–	–	–	–	–	–	48.19	36.144	24.096	–	–	–
HPMC	–	–	–	–	–	–	–	–	–	48.19	36.144	24.096
Liquid Vehicle	36.144	36.144	36.144	36.144	36.144	36.144	36.144	36.144	36.144	36.144	36.144	36.144
Primojel	1.205	1.205	1.205	1.205	1.205	1.205	1.205	1.205	1.205	1.205	1.205	1.205
Mg. Stearate	0.602	0.602	0.602	0.602	0.602	0.602	0.602	0.602	0.602	0.602	0.602	0.602
Tablettose-80	0	13.26	26.506	0	13.26	26.506	0	13.26	26.506	0	13.26	26.506

Quantities are given as %w/w.

HPMC; Hydroxypropyl methylcellulose.

Liquid Vehicle; Non-volatile solvent system consisting of propylene glycol and water in 1:1.

Primejel; Sodium starch glycolate.

Mg. stearate; Magnesium stearate.

Tablettose-80; Granular lactose.

### Evaluation of liquisolid compacts

All the formulations of liquisolid compacts were evaluated at both pre compression and post compression level.

#### Pre compression evaluation

Prior to compression, powder blend was evaluated for various parameters related to flow such as bulk density, tapped density, angle of repose, Carr’s index and Hausner ratio, according to their official methods [28]. All determinations were made in triplicate and results were presented as mean ± SD (n = 3).

FTIR spectra of pure drug and final formulation were recorded using FTIR spectrophotometer (FTIR Prestige, Shimadzu, Japan) equipped with IR Solutions version 1.10 software. KBr pellet method was used for sample preparation. Sample (2% w/w) was mixed with KBr, pulverized spectra were recorded in 400–4000 cm^-1^ spectral region at resolution of 8 cm^-1^.

#### Post compression evaluation

Liquisolid compacts were evaluated for various parameters related to physical characteristics, mechanical strength, disintegration behavior and drug release as follows;

*Physical characteristics of liquisolid compacts*. Physically liquisolid compacts were evaluated for their appearance and surface defects. Weight variation of liquisolid compacts was determined according to official monograph [28] by measuring weight of twenty compacts, individually and collectively. Thickness of liquisolid compacts (n = 10) was measured by digital tablet hardness and thickness tester (Pharmatest, Hamburg, Germany) and its mean and standard deviation were calculated.

*Drug content of liquisolid compacts*. Drug content of Liquisolid compacts was determined according to USP [28]. Liquisolid compacts (n = 10) were randomly selected from each formulation and crushed to fine powder. Powder equivalent to the unit dose of clopidogrel was transferred to a volumetric flask (100 mL), containing methanol, shaken for 30 min and volume was made up to the mark with mobile phase. Solution was filtered and injected (10 μL) to HPLC. Mobile phase consisted of mixture of phosphate buffer (pH 6.8) and acetonitrile in 3:1 by volume, pumped at a flow rate of 1.3 mL/min. Detector wavelength was set at 220 nm. Separation was carried out on an L57 (4.6 mm x 15 cm) column.

A standard solution of same concentration was prepared using methanol as solvent and analyzed under the same conditions. Drug content was calculated by comparing peak area of the sample solution with that of standard solution, as follows;

$$Percent\;Drug\;Content=\frac{\text{A}\;\text{sample}}{\text{A}\;\text{standard}}\times 100$$ (4)

Where

A sample = Peak area of sample solution
A standard = Peak area of standard solution

*Mechanical strength of liquisolid compacts*. Mechanical strength of liqui-solid compacts was estimated on the basis of crushing strength, specific crushing strength, tensile strength and friability. All the parameters were determined in accordance with the official monograph [28].

Liquisolid compacts (n = 10) were randomly selected from each formulation; their crushing strength was measured by digital hardness and thickness tester (PharmaTest, Hamburg, Germany) and mean value was taken. Tensile strength and specific crushing strength of liquisolid compacts were calculated using following equations;

$$Ts=\frac{2\text{F}}{\pi \text{TD}}$$ (5)

$$\tau =\frac{\text{F}}{\text{TD}}$$ (6)

Where

T_s_ = Tensile strength of liquisolid compacts (kg/mm^2^)
τ = Specific hardness of liquisolid compacts (Kg/mm^2^)
F = Crushing strength of liquisolid compacts (Kg)
T = Thickness of liquisolid compacts (mm)
D = Diameter of liquisolid compacts (mm)
π = Constant and its value is 3.143

Friability of liquisolid compacts was determined using a single drum friabilator (Faisal Engineering, Pakistan) as per official compendia [28].

*Disintegration time*. Disintegration time of liquisolid compacts was determined according to USP [28] using USP tablet disintegration testing apparatus (PharmaTest, Hamburg, Germany). Distilled water at 37 ± 2°C was used as disintegration medium. Liquisolid compacts (n = 6) were randomly selected and subjected to disintegration test. Mean of six determinations was taken as disintegration time and results were presented as mean ± S.D (n = 6).

*In vitro drug release*. *In vitro* dissolution studies were performed using USP apparatus-II (paddle type) at 50 rpm and 0.1N HCl (900 mL) as dissolution media at 37 ± 2°C [16]. The samples were withdrawn at different time intervals, filtered and analyzed for drug content by HPLC. At each sampling point, analysis was made in triplicate and mean value was taken.

*Comparison of dissolution profiles*. Comparison of dissolution profile was based on similarity factor (*f*_*2*_), dissimilarity factor (*f*_*1*_) and dissolution efficiency (D.E.). Dissimilarity and similarity factor were calculated using Eqs 7 and 8, respectively [30, 31].

$$f1=\frac{\sum [Rt-Tt]}{\sum Rt}x100$$ (7)

$$f_{2}=50\;\text{x}\;\text{log}\;\left\{{\left[1+\left(1/\text{n}\right)\;\sum {\left({\text{R}}_{\text{t}}-\;{\text{T}}_{\text{t}}\right)}^{2}\right]}^{-0.5}\text{x}\;100\right\}$$ (8)

Where

R_t_ = Dissolution rate of standard product at time “t”
T_t_ = Dissolution rate of test product at time t

Control formulation prepared by direct compression technique was used as standard for comparison of dissolution profiles. The “*f*_*2*_*”* value of 50 or greater ensures sameness or equivalence of the two curves and also the performance of the two products.

## Results and discussion

### Solubility and stability of clopidogrel in non volatile solvent system

According to biopharmaceutical classification system, clopidogrel is a class-II drug and is practically insoluble in water [32]. However its solubility increases by heating. CLP is more soluble at acidic pH (HCl buffer; pH 2 = 296.89 ± 3.49 μg/L). Increase in pH cause decrease in solubility (101.5 ± 3.72 μg/L at pH 6.8).

Solubility of CLP was checked in different non-volatile solvents/ solvent systems and selection was made on the basis of higher solubility. CLP was highly soluble in mixture of propylene glycol and water (2:1) by volume, as shown in Table 3. Solubility of clopidogrel decreased by inclusion of tween-60 and it was in accordance with the reported literature [33]. Tween-60 is a surfactant and usually increases water solubility. Due to its alkaline nature, pH of aqueous solution of tween-60 is high. CLP shows pH dependent solubility, which decreases by increasing pH. It was the reason for decreased solubility of CLP in solvent systems containing tween-60. Solubility of CLP in different nonvolatile solvents and their mixture is summarized in Table 3.

**Table 3:** Solubility of clopidogrel in different nonvolatile solvent and their mixture.

Solvent	Solubility (mg/L)
Water	97.29 ± 1.08
Glycerin	136 ± 0.92
PG	582.67 ± 1.19
PG + Water (1:1)	239.80 ± 0.95
PG + water (1:2)	149.00 ± 1.02
PG + water (2:1)	2986.27 ± 0.79
PG + water +Tween-60 (0.5%)	1145.00 ± 1.28
Glycerin + Water (1:1)	109.56 ± 1.01
Glycerin + Water (1:2)	98.71 ± 0.27
Glycerin + Water (2:1)	108.90 ± 0.91
Glycerin + Water + Tween-60 (0.5%)	92.86 ± 0.67

Results are presented as Mean ± SD (n = 3).

PG; Propylene Glycol.

**Fig 2:**
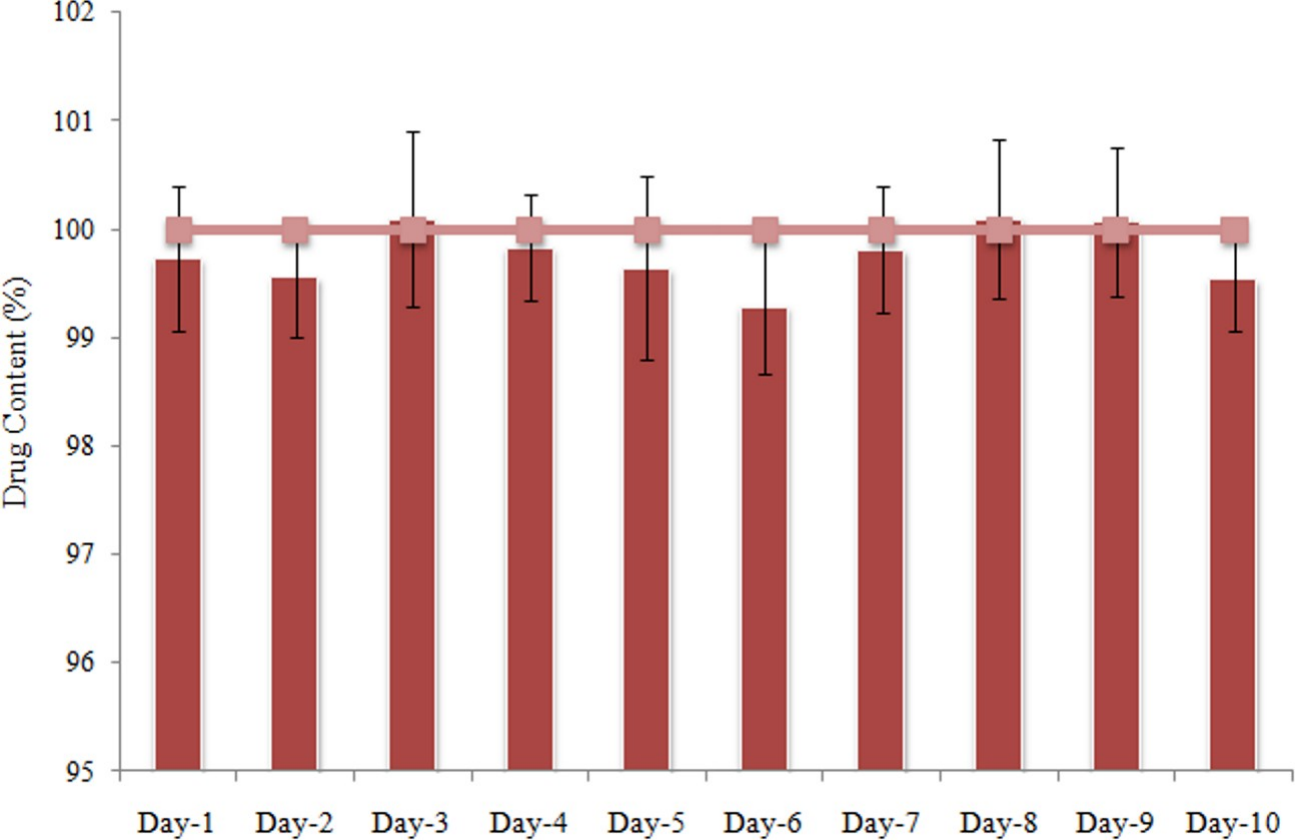
Drug content of clopidogrel solutions prepared in liquid vehicle (PG + water, 2:1 v/v) at specified time intervals (24 h) after storage at 45 ± 5°C and 75 ± 5% relative humidity.

Stability of CLP solution prepared in the selected liquid vehicle (propylene glycol + water) was evaluated for ten days at stressed conditions (45 ± 5°C and 75 ± 5% R.H.). Drug content was determined on daily basis and compared with initial results (day-1). Clopidogrel content of the solution/ dispersion remained un-affected throughout the study period and no change was observed in physical characteristics (precipitation, color and odor) of the solution. Fig 2 presents CLP content of the solution at different sampling points.

### Pre compression evaluations of liquisolid compacts

In liquisolid compacts, drug is incorporated in liquid form (solution) which can affect flow and compressibility. Results of flowability, angle of repose, Carr’s index and Hausner ratio indicated better flow characteristics (Table 4) in comparison with control formulation (prepared by direct compression technique). This shows that the presence of liquid vehicle in liquisolid formulation can improve flowability of powders, attributed to their granulating effect. In addition, the liquid vehicle containing the drug (liquid medication) can initially be absorbed by the carrier surfaces followed by covering the carrier surfaces by the fine coating particles (colloidal silicon dioxide) to yield granules with high flowability.

**Table 4:** Pre Compression evaluation of liquisolid compacts containing clopidogrel.

Formulation Code	Bulk Volume (mL)	Tapped Volume (mL)	Bulk Density (g/mL)	Tapped Density (g/mL)	Hausner Ratio	Carr’s Index (%)	Angle of Repose (o)
Control Formulation	25.00	16.59	0.33	0.49	1.48	32.65	35.80
LSC-01	25.00	20.00	0.57	0.71	1.25	20.00	33.76
LSC-02	25.00	17.50	0.43	0.61	1.41	29.50	30.09
LSC-03	25.00	19.00	0.42	0.55	1.31	23.92	29.82
LSC-04	25.00	19.50	0.59	0.76	1.28	21.99	34.66
LSC-05	25.00	19.50	0.61	0.78	1.28	22.00	30.93
LSC-06	25.00	18.00	0.48	0.67	1.39	28.00	30.17
LSC-07	25.00	17.50	0.43	0.61	1.43	30.00	32.42
LSC-08	25.00	17.50	0.43	0.62	1.43	29.99	29.13
LSC-09	25.00	17.00	0.43	0.63	1.47	32.00	31.59
LSC-10	25.00	17.60	0.34	0.48	1.41	29.17	30.69
LSC-11	25.00	19.00	0.45	0.60	1.00	23.99	32.37
LSC-12	25.00	19.00	0.52	0.67	1.32	23.99	36.31

Data is presented as mean of triplicate determination (n = 3).

Data has been rounded to two digits after decimal point.

**Fig 3:**
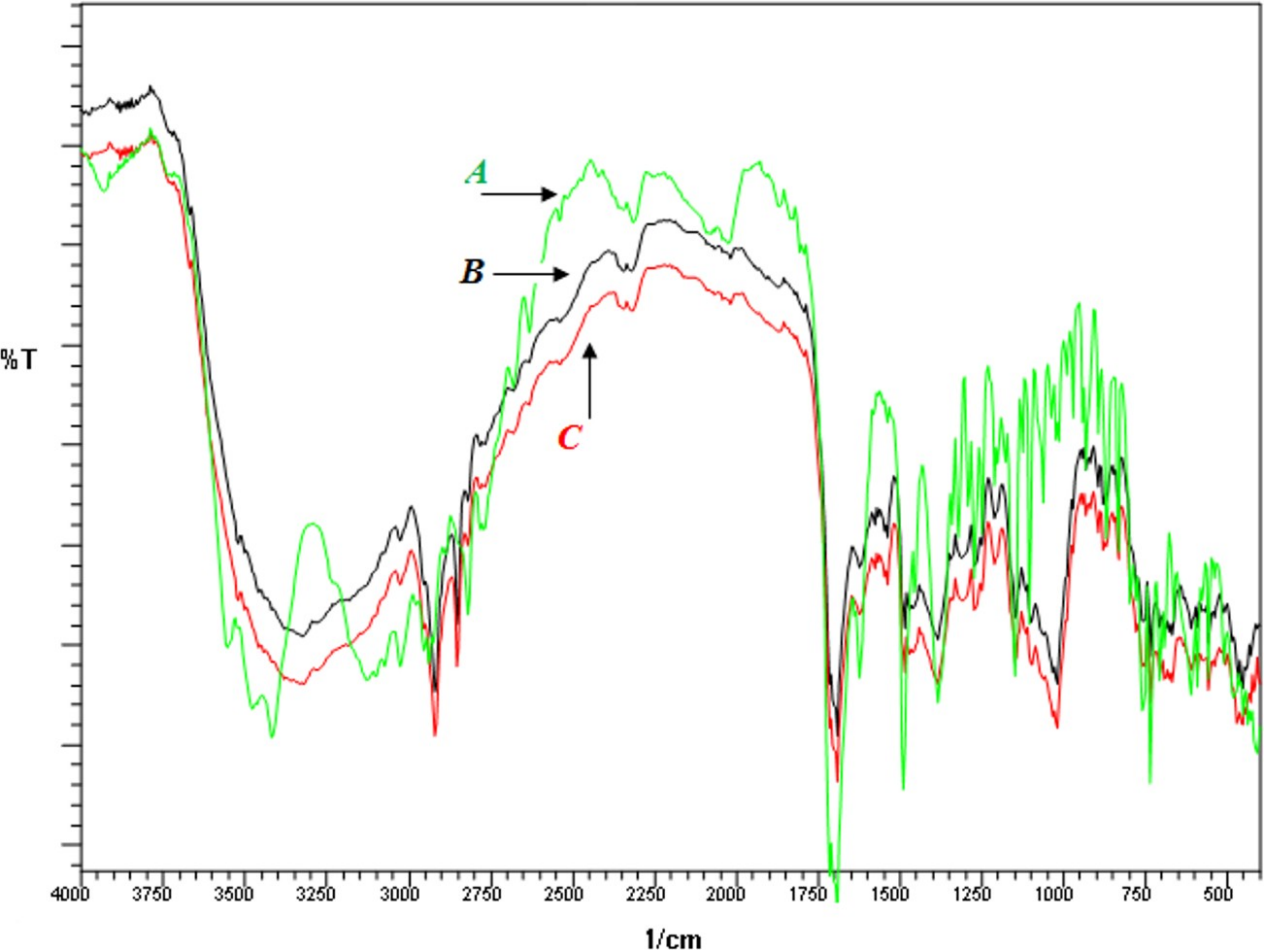
FTIR spectra of clopidogrel (A), excipients (B) and final formulation containing clopidogrel and excipients (C).

FTIR spectra is the finger print for any compound as it represents its different bonds, functional groups and their vibrations. Chemical interactions result in breaking and formation of new bond, changing chemical nature of the compound. Any change in FTIR spectra of a compound predicts changes in its chemical structure and indicates degradation or interaction of drug with excipients. It has been reported that presence of characteristic peaks in an FTIR spectra of a compound rules out chances of interactions [33]. FTIR spectra showed that all the characteristic peaks were present, indicating the absence of any chemical interaction. FTIR spectra of pure CLP (API), excipients (without API) and final formulation (API + excipients) are presented in Fig 3.

Better flow characteristics were obtained with the formulation containing micro crystalline cellulose (LCS-03). Because of its rough surface, micro crystalline cellulose showed better adsorbing capacity and the resultant powder was drier as compared with starch.

Formulations containing starch were expected to show better flow characteristic due to smaller particle size and larger surface area. But their flow characteristics were poor in comparison with micro crystalline cellulose because its surface was smooth and had little adsorbing capacity.

### Post compression evaluation of liquisolid compacts

#### Physical characteristics of liquisolid compacts of clopidogrel

Liquisolid compacts were compressed using round plane punches (12 mm diameter) with beveled edges and bisection line on one side. Compression weight of liquisolid compacts was 830 mg and at least 300 tablets were compressed for each formulation. Tablets from all the formulations were free of physical defects and were smooth and shiny, indicating that liquid vehicle was properly adsorbed on the carrier material. Weight variation was determined according to USP and was within the official limits, as shown in Table 5. Low values of weight variation indicated proper flow of the powder during compression. Drug content of liquisolid compacts was above 99% which was within the official limits (90–110%), confirming proper mixing of the drug with excipients.

**Table 5:** Physical parameters of liquisolid compacts containing clopidogrel.

Formulation Code	Average Weight*† (mg)	Weight Variation (%)	Thickness* (mm)	Drug Content** (%)
Control Formulation	839.30	±3.98	5.11 ± 0.21	100.08 ± 0.39
LSC-01	838.07	±3.37	5.08 ± 0.19	99.01 ± 0.24
LSC-02	840.50	±2.43	4.89 ± 0.24	99.50 ± 3.17
LSC-03	834.93	±3.79	4.65 ± 0.41	99.31 ± 1.29
LSC-04	837.48	±3.83	5.13 ± 0.19	99.76 ± 2.31
LSC-05	839.50	±2.68	5.01 ± 0.74	99.81 ± 1.09
LSC-06	836.81	±2.49	5.13 ± 0.28	99.05 ± 1.73
LSC-07	839.79	±2.24	5.75 ± 0.37	99.27 ± 1.19
LSC-08	834.53	±2.38	5.61 ± 0.37	100.96 ± 1.59
LSC-09	841.50	±3.05	5.07 ± 0.15	99.92 ± 1.60
LSC-10	843.57	±3.22	4.97 ± 0.48	99.17 ± 1.18
LSC-11	837.39	±1.37	5.05 ± 0.52	101.00 ± 1.52
LSC-12	838.26	±2.71	4.92 ± 0.63	99.15 ± 1.09

Data has been rounded to two digits after decimal point.

*Data is presented as mean± SD (n = 10).

**Data is presented as mean± SD (n = 3).

#### Mechanical strength of liquisolid compacts

Mechanical strength of liquisolid compacts was determined on the basis of their crushing strength, specific crushing strength, tensile strength and friability, determined according to USP [28]. Crushing strength of the formulations was in the range of 6–11 kg. Friability of liquisolid compacts was in the range of 0.15–0.43%, indicating acceptable resistance to withstand handling. One of the main hurdles with liquisolid compacts is their poor compressibility which makes them difficult to produce robust and strong tablets [34]. Table 6 shows that liquisolid compacts are superior to the control formulation in terms of mechanical strength.

**Table 6:** Mechanical strength of liquisolid compacts of clopidogrel.

Formulation Code	Crushing Strength (kg)	Specific Crushing Strength (kg/mm^2^)	Tensile Strength (kg/mm^2^)	Friability (%)
Control Formulation	7.21 ± 0.38	0.0739	0.1162	0.49
LSC-01	8.39 ± 0.91	0.0876	0.1376	0.15
LSC-02	7.59 ± 0.46	0.0823	0.1293	0.15
LSC-03	10.21 ± 2.05	0.1163	0.1828	0.19
LSC-04	8.71 ± 0.36	0.0900	0.1415	0.25
LSC-05	8.88 ± 0.61	0.0940	0.1477	0.15
LSC-06	7.19 ± 0.79	0.0743	0.1168	0.43
LSC-07	8.04 ± 0.53	0.0742	0.1165	0.19
LSC-08	6.25 ± 0.34	0.0591	0.0928	0.35
LSC-09	8.23 ± 1.40	0.0861	0.1353	0.15
LSC-10	9.47 ± 2.92	0.1010	0.1588	0.29
LSC-11	8.11 ± 0.30	0.0852	0.1338	0.28
LSC-12	10.4 ± 2.36	0.1121	0.1762	0.15

*Data is presented as mean± SD (n = 10).

** Calculations were made on the basis of mean crushing strength and thickness of liquisolid compacts.

Liquid vehicle containing the drug (liquid medication) can initially be absorbed by the carrier surfaces followed by covering the carrier surfaces by the fine coating particles (colloidal silicon dioxide) to yield granules with high mechanical strength. Presence of PVP and HPMC could have remarkable effect on the mechanical properties of liquisolid tablets. In conclusion, it can be concluded that the liquisolid formulations showed superior mechanical strength than the control formulation (prepared by direct compression technique) which makes the liquisolid compact approach appealing to industry.

### Disintegration behavior of liquisolid compacts

**Fig 4:**
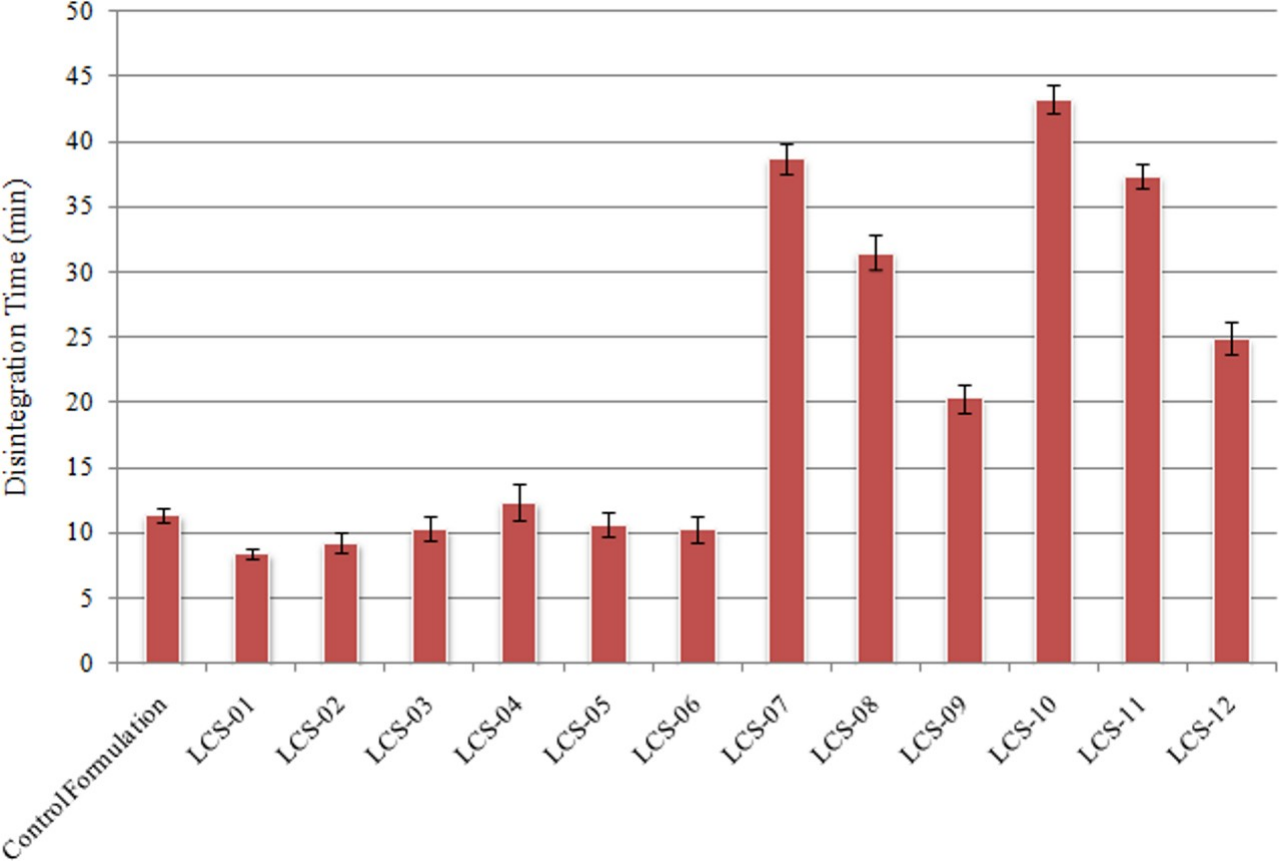
Disintegration time of liquisolid compacts of Clopidogrel. Disintegration time was determined in accordance with USP, using purified water as disintegration media at 37 ± 2°C.

Disintegration time was determined according to the official monograph [28], using purified water as disintegration medium, at 37 ± 2°C. Formulations containing MCC and starch showed relatively rapid disintegration (<15 min). Comparison of disintegration time of control formulation (11.39 ± 0.58 min) and liquisolid compacts showed better disintegration of liquisolid compacts containing MCC and starch (Fig 4). Disintegration of liquisolid compacts was controlled by the nature and quantity of carrier material. Liquisolid compacts containing MCC and starch as carrier material showed smaller disintegration time while those containing PVP and HPMC had longer disintegration time. The formulation LCS-10 had highest disintegration time (43.24 ± 1.05 min; n = 6) due to highest quantity of HPMC (48.19% w/w).

### *In vitro* release of clopidogrel from liquisolid compacts

**Fig 5:**
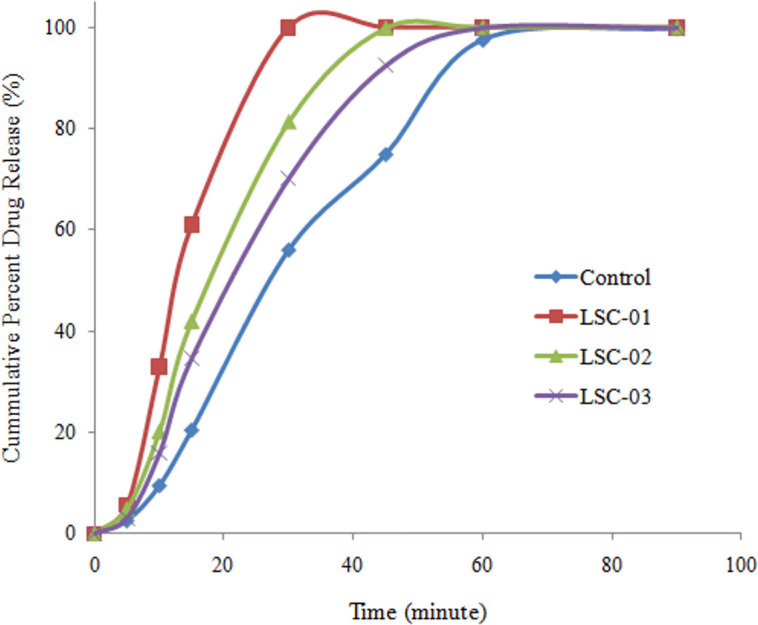
Drug release from liquisolid compacts containing different concentrations of MCC.

**Fig 6:**
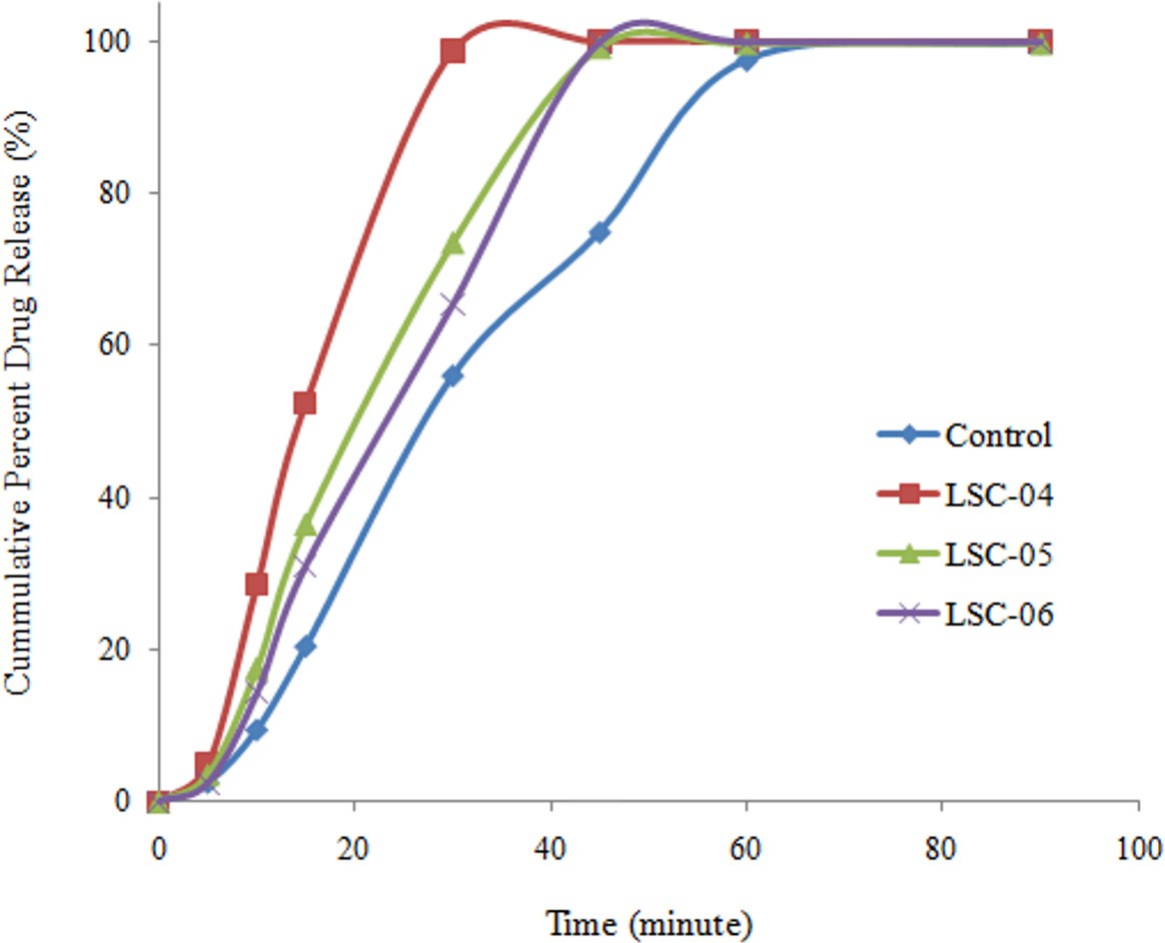
Drug release from liquisolid compacts containing different concentrations of starch.

Dissolution rate from liquisolid compact was determined in accordance with the official compendia, using 0.1N HCl (900 mL) as dissolution media. *In-vitro* drug release from liquisolid compacts was controlled by nature and quantity of carrier material. Highest release was observed with starch maize and micro crystalline cellulose while inclusion of PVP and HPMC resulted in sustained drug release. Dissolution profiles of liquisolid compacts were compared with control formulation on the basis of dissimilarity (*f*_*1*_) and similarity factor (*f*_*2*_) which showed that there was no similarity between the dissolution profiles as the values of *f*_*1*_ was larger while *f*_*2*_ had a small value for all the formulations. Liquisolid compact is a versatile technique as it can modify the drug release in both ways i.e. can enhance release rate resulting in rapid availability of the drug at the absorption site and can sustain drug release for longer period of time. Hydrophilic carrier results in better water absorption and increases drug-water interaction which leads to better solubility and enhanced dissolution rate. As shown in Figs 5 and 6, microcrystalline cellulose and starch maize resulted in faster drug release. Furthermore, increase in the quantity of the hydrophilic carrier, increased dissolution rate in a concentration dependent manner.

Comparison of dissolution profiles was made on the basis of burst effect (amount of drug released during initial 15 min). Highest burst release was observed with microcrystalline cellulose (61.03 ± 1.39%; n = 6) which was 66.44% higher than control formulation. Similarly, starch showed 60.98% higher burst release than control formulation. It indicated that using hydrophilic carrier material dissolution rate can be significantly enhanced by liquisolid technique. Furthermore, in liquisolid compact maximum drug release was observed in relatively shorter time as compared with control formulation.

In the present study two gel forming polymers PVP and HPMC were used for sustaining the drug release. Both the material forms a matrix which forms gel upon contact with dissolution media [35]. Dissolution media penetrates the inner portions of matrix, layer by layer and drug is released in a controlled manner for longer period of time. Rate of the drug release from matrix is controlled by;

- Diffusion of drug through gel layer
- Erosion of gel layer

In comparison to conventional sustained release matrix tablets, liquisolid compacts has the advantage that drug is mixed with matrix forming carrier in solution/ dispersion form and is uniformly distributed throughout the carrier material which results in a uniform drug release [36].

**Fig 7:**
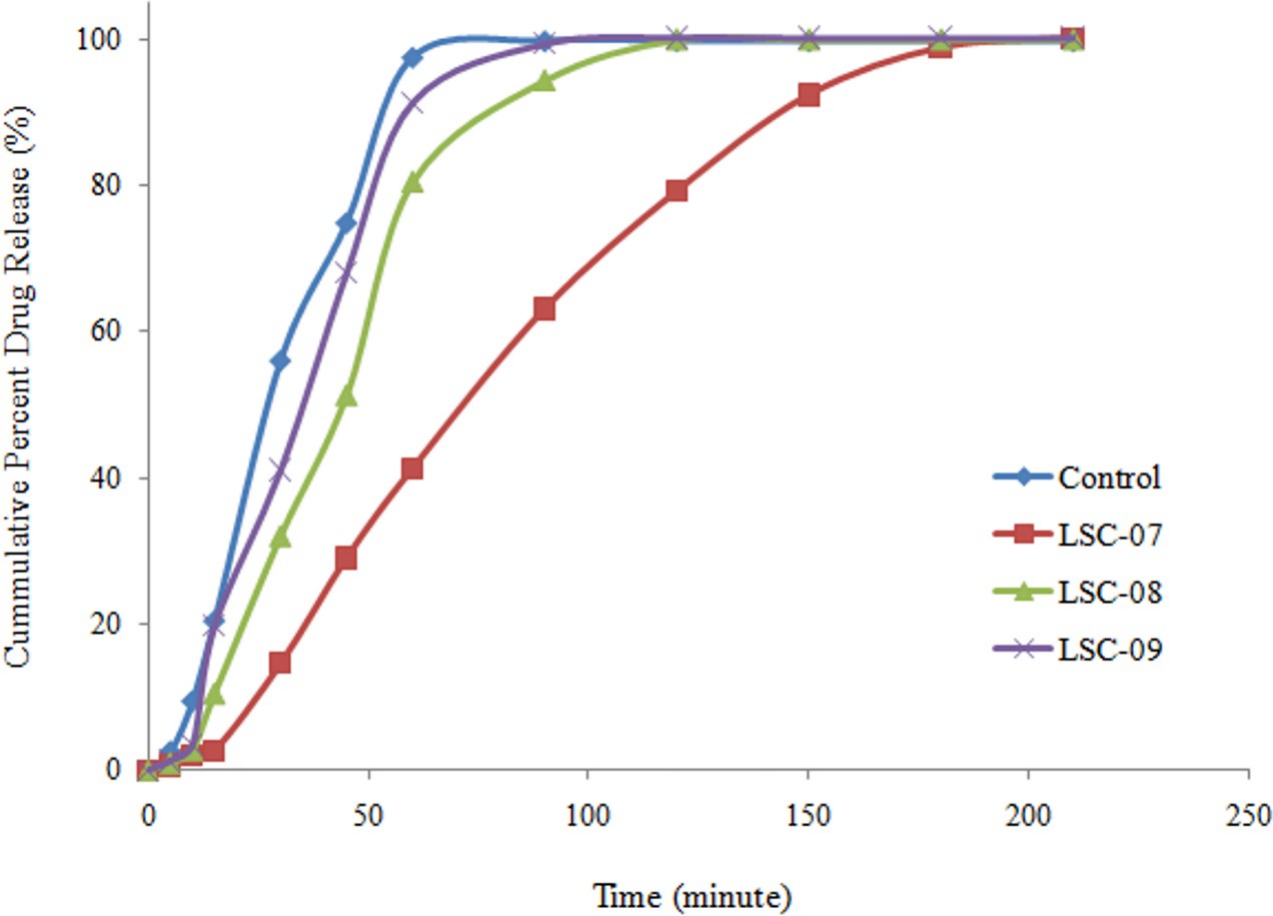
Drug release from liquisolid compacts containing different concentrations of PVP.

**Fig 8:**
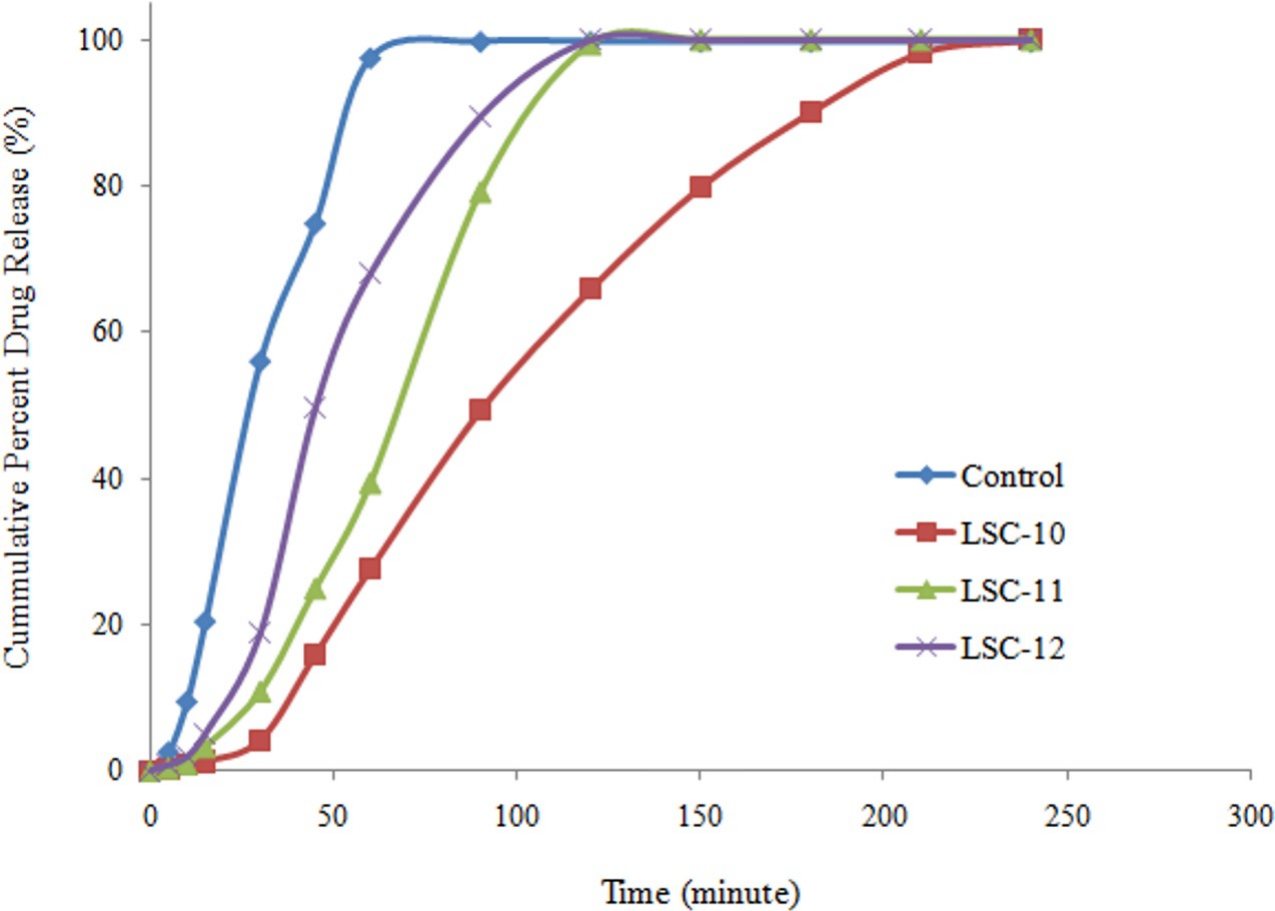
Drug release from liquisolid compacts containing different concentrations of HPMC.

In the present study an attempt was made to investigate the effect of different concentrations of PVP and HPMC on drug release from liquisolid compacts and their dissolution profiles were compared to that of conventional tablets, prepared by direct compression technique. The effect of incorporating PVP in liquisolid formulation on dissolution rate of clopidogrel is shown in Fig 7. Higher concentration of PVP (48.19%, w/w) decreased the percentage of drug release which was 86.91% of the control formulation. Furthermore, drug release was prolonged for 3.5 h. decrease in drug release was dependent up on the concentration of PVP. Decrease in quantity of PVP resulted in relatively faster release and maximum drug released within 2 h at 25.97%, w/w concentration. Dissolution profile of CLP from liquisolid compact containing HPMC is shown in Fig 8. Like PVP it also retarded the drug release for longer period of time. Maximum drug release was observed within 4 h at higher concentration (48.19% w/w) of HPMC.

**Fig 9:**
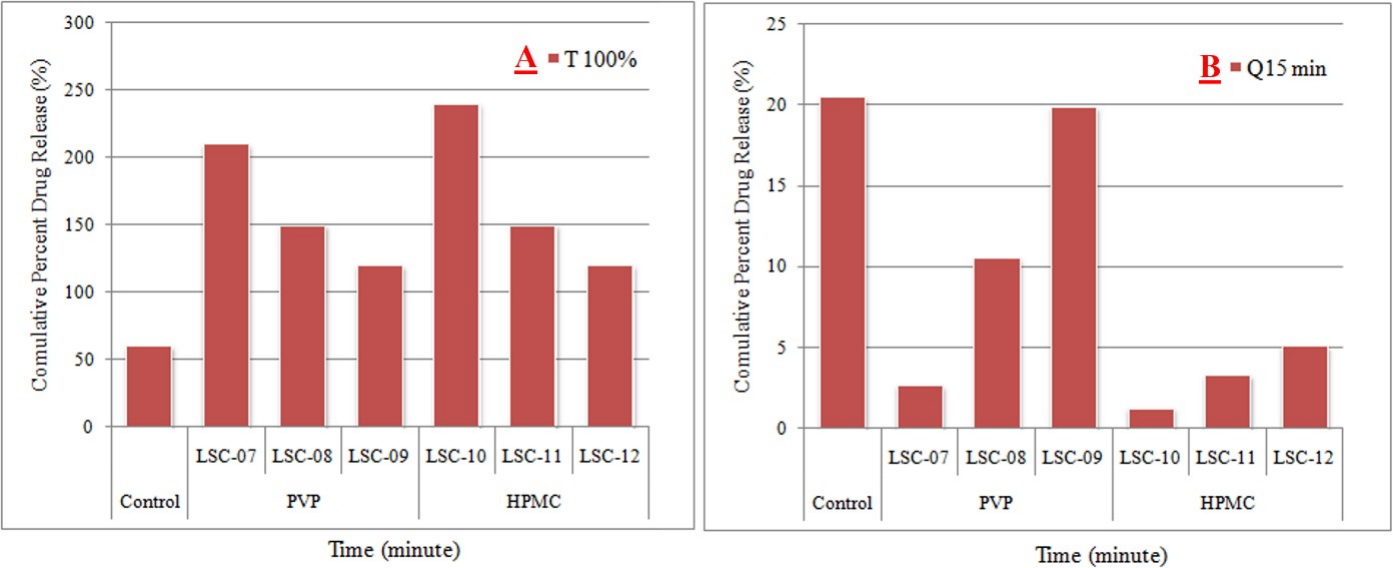
Comparison of time taken for maximal drug release (T100%) and amount of drug release during initial 15 min (Q15min) from liquisolid compacts containing different concentrations of PVP and HPMC, with control formulation (tablets prepared by direct compression technique.

Comparison of drug release during initial 15 min showed that 93.8% decrease in drug release was observed using HPMC as carrier material in comparison with control formulation. Comparison of PVP and HPMC showed that HPMC retarded drug release for relatively longer time and its release rate was slower at all the tested levels as shown in Fig 9.

## Conclusion

Aim of the study was to evaluate the effect of carrier material on dissolution rate of poor water soluble drug (clopidogril) by formulating as liquisolid compacts. It is concluded from the study that modifications in drug release are controlled by nature and quantity of the carrier material. Clopidogril is a poor water soluble drug and its dissolution rate was significantly enhanced by formulating as liquisolid compact, using hydrophilic carriers (MCC and starch). Inclusion of gel forming polymeric carrier (PVP and HPMC) resulted in sustaining the drug release. It is concluded from results that both fast release and sustained release of clopidogrel can be achieved by proper selection of carrier material.
